# An Unusual Case of Rapidly Progressive Hyperbilirubinemia

**DOI:** 10.1155/2013/284029

**Published:** 2013-10-29

**Authors:** Kimberly M. Thornton, Michael F. Nyp, Lejla Music Aplenc, Gary L. Jones, Shannon L. Carpenter, Erin M. Guest, Steven M. Shapiro, Winston M. Manimtim

**Affiliations:** ^1^Division of Neonatology-Perinatology, Children's Mercy Hospitals and Clinics, 2401 Gillham Road, Kansas City, MO 64108, USA; ^2^School of Medicine, University of Missouri-Kansas City, 2411 Holmes Road, Kansas City, MO 64108, USA; ^3^Division of Pathology, Children's Mercy Hospitals and Clinics, 2401 Gillham Road, Kansas City, MO 64108, USA; ^4^Division of Hematology-Oncology, Children's Mercy Hospitals and Clinics, 2401 Gillham Road, Kansas City, MO 64108, USA; ^5^Division of Neurology, Children's Mercy Hospitals and Clinics, 2401 Gillham Road, Kansas City, MO 64108, USA

## Abstract

We present an unusual case of hyperbilirubinemia with rapid early progression leading to bilirubin encephalopathy in a term neonate. Despite early recognition and intervention, the total serum bilirubin reached a maximum level of 39 mg/dL at 32 hours of life. Prior to an emergent exchange transfusion, the patient's diagnostic evaluation was significant for Coombs-negative microangiopathic hemolytic anemia and thrombocytopenia. Further testing revealed a deficiency of ADAMTS13 protein, or von Willebrand factor-cleaving protease, a finding diagnostic of congenital thrombotic thrombocytopenic purpura, or Upshaw-Schulman syndrome. This rare disease is often misdiagnosed, especially in the newborn period.

## 1. Introduction

Hyperbilirubinemia is one of the most common reasons for admission to an intensive care nursery. Despite a systematic approach towards prevention, bilirubin encephalopathy still occurs and remains as a neonatal emergency requiring early intervention to prevent permanent neurologic sequelae. Here we report an atypical presentation of bilirubin encephalopathy due to congenital thrombotic thrombocytopenic purpura (TTP).

## 2. Case Presentation

A 40-week gestation, 3.1 kg, Hispanic male infant was born via spontaneous vaginal delivery to a 22-year-old gravida 4 para 3 spontaneous abortion 1 mother without significant past medical history. Maternal blood type was O+ and antibody screen was negative. Other maternal prenatal labs were unremarkable. There was no known history of jaundice, anemia, or hematologic disorders in family members. Apgar scores were 7 and 8 at 1 and 5 minutes, respectively. At 4 hours of age, the infant was noted to have a hematoma at his vitamin K intramuscular injection site. He developed jaundice at <24 hours of age, with a total serum bilirubin (TSB) of 33.3 mg/dL at 29 hours of age. High intensity phototherapy was promptly initiated and he was transferred to a level III neonatal intensive care unit.

**Figure 1:**
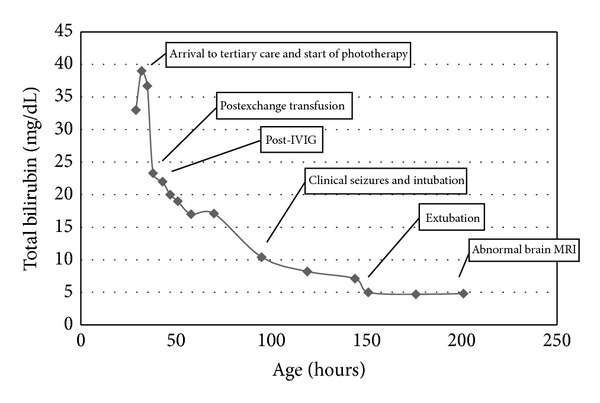
Graph of the patient's total bilirubin values (mg/dL) and age (hours) with pertinent clinical events.

Laboratory evaluation revealed infant blood type O+, negative direct antibody test, mild anemia (hemoglobin 11.6 gm/dL), thrombocytopenia (26,000), elevated serum creatinine (1.3 mg/dL), elevated aspartate aminotransferase (248 units/L), and elevated reticulocyte count (4.9%). All other labs were within normal limits, including the white blood cell count and coagulation studies (prothrombin time, partial thromboplastin time, and fibrinogen). The TSB was eventually as high as 39 mg/dL at 32 hours of life. On physical exam, he was markedly jaundiced, irritable, and hypertonic. He was also noted to have a high-pitched cry and intermittent opisthotonic posturing. At 35 hours of age, he underwent double-volume exchange transfusion with reconstituted packed RBCs. After-exchange, the TSB was 23.3 mg/dL and declined steadily thereafter (Figure 1).

**Figure 2:**
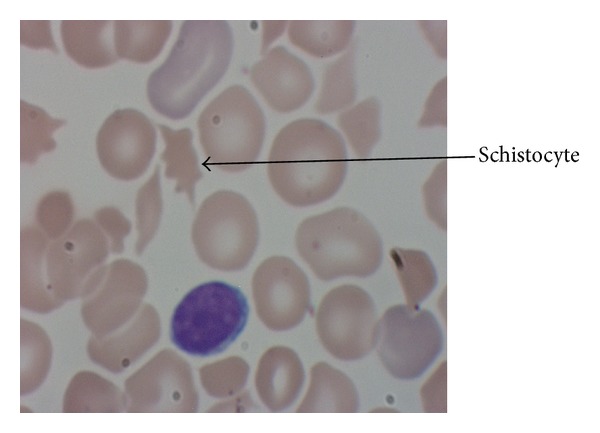
The patient's peripheral blood smear showing microangiopathic hemolytic anemia.

Following the exchange transfusion, he developed symptoms of encephalopathy including seizures and persistent apnea requiring mechanical ventilation for two days. EEG confirmed multifocal seizures and diffuse encephalopathy. Brain MRI at 8 days showed T1-weighted hyperintensity of the globus pallidus. Peripheral blood smear showed microangiopathic hemolysis, suggestive of TTP among other diagnoses (Figure 2). The ADAMTS13 (a disintegrin and metalloproteinase with a thrombospondin type 1 motif, member 13) enzymatic activity was measured using the FRETS-VWF73 substrate, a synthetic 73-amino-acid peptide. Cleavage of the substrate by ADAMTS13 causes it to fluoresce over time, with patients deficient in the ADAMTS13 enzyme showing a decrease in fluorescence compared with controls. Our patient was confirmed to be deficient in an enzyme activity of <5%. Gene sequencing analysis showed a heterozygous missense mutation in the ADAMTS13 gene [c.304C>T (p.Arg102Cys)]. 

Because hyperbilirubinemia can interfere with the FRETS-VWF73 substrate assay, the ADAMTS13 level was repeated at around 5 months of age, when the child was not in an acute hemolytic state. Results of the testing again showed an enzyme activity level of <5%. At 8 months, he had auditory predominant kernicterus [1], mild truncal hypotonia, and impaired upward gaze. His sensorimotor development was otherwise normal for age. Diagnostic auditory testing at the time revealed a severe auditory neuropathy spectrum disorder. The patient's neurologic deficits have improved over time, but he continues to have significant hearing loss. He receives regular prophylactic FFP infusions and is being considered for cochlear implants.

## 3. Discussion

TTP is a disease in which microthrombi form in multiple small vessels throughout the body leading to signs and symptoms of organ ischemia. The normal coagulation process involves von Willebrand factor (vWF), a large multimeric protein which binds platelets to areas of intravascular endothelial cell damage. In normal hemostasis, the function of ADAMTS13 protease is to cleave vWF multimers [2]. When ADAMTS13 activity is deficient, ultra-large vWF multimers accumulate, leading to the formation of platelet-rich intravascular microthrombi [2]. These micro-thrombi, in turn, cause damage to circulating red blood cells as well as ischemia to vital organs.

Acquired TTP, the more common form, is usually an autoimmune disease caused by antibodies against ADAMTS13 protein. The rare congenital form of disease, also known as Upshaw-Schulman syndrome, is caused by a mutation of the gene coding for ADAMTS13 found on chromosome 9q34 and follows an autosomal recessive pattern of inheritance [3]. The mutation results in a deficiency of the protein itself or a decrease in the functionality of the protein. Unlike cases of immune-mediated hemolysis, congenital TTP may be treated with transfusions of fresh frozen plasma to replace the missing protein.

Multiple gene mutations are associated with severe deficiency of ADAMTS13 activity, and the cause of variable phenotypic expression is not yet understood [4]. Very few cases of congenital TTP have been recognized during the newborn period, and, upon review of cases diagnosed in later childhood, some had a history of severe hyperbilirubinemia after birth requiring double-volume exchange transfusion [2, 4, 5].

The classic symptoms of chronic bilirubin encephalopathy, or kernicterus, include dystonic or athetoid cerebral palsy, auditory neuropathy spectrum disorder with or without hearing loss, impaired upward gaze, and tooth enamel dysplasia [1]. Brain MRI shows hyperintensity of the globus pallidus and, possibly, subthalamic nuclei on T1-weighted imaging when performed in the first weeks after birth, with hyperintensity in these areas on T2-weighted imaging performed later [1].

Our patient's brain MRI findings are more consistent with bilirubin toxicity rather than a microvascular ischemic event, which could also be present in a child with congenital TTP. Our patient's genetic testing has only revealed a heterozygous mutation of the ADAMTS13 gene, but it is postulated that the other chromosome either has a mutation in a noncoding region or a mutation affecting protein processing that has yet to be identified. There have been other cases reported in the literature with significant disease and only one allele affected [2]. It is important to note that while genetic testing can be informative, low ADAMTS13 activity is considered diagnostic of congenital TTP.

In conclusion, congenital TTP is a rare disease but should be considered in any case of hyperbilirubinemia occurring in conjunction with thrombocytopenia and hemolytic anemia. This case points out the importance of obtaining a peripheral smear in cases of strikingly high TSB levels.
